# Equine Embryonic Stem Cell-Derived Tenocytes are Insensitive to a Combination of Inflammatory Cytokines and Have Distinct Molecular Responses Compared to Primary Tenocytes

**DOI:** 10.1007/s12015-024-10693-8

**Published:** 2024-02-24

**Authors:** Emily J. Smith, Ross E. Beaumont, Jayesh Dudhia, Deborah J. Guest

**Affiliations:** Department of Clinical Sciences and Services, The Royal Veterinary College, Hawkshead Lane, North Mymms, Hatfield, Herts AL9 7TA UK

**Keywords:** Horse, Cytokine, Tendon, Inflammation, NF-κB, Embryonic Stem Cell

## Abstract

Tissue fibrosis following tendon injury is a major clinical problem due to the increased risk of re-injury and limited treatment options; however, its mechanism remains unclear. Evidence suggests that insufficient resolution of inflammation contributes to fibrotic healing by disrupting tenocyte activity, with the NF-κB pathway being identified as a potential mediator. Equine embryonic stem cell (ESC) derived tenocytes may offer a potential cell-based therapy to improve tendon regeneration, but how they respond to an inflammatory environment is largely unknown. Our findings reveal for the first time that, unlike adult tenocytes, ESC-tenocytes are unaffected by IFN-γ, TNFα, and IL-1β stimulation; producing minimal changes to tendon-associated gene expression and generating 3-D collagen gel constructs indistinguishable from unstimulated controls. Inflammatory pathway analysis found these inflammatory cytokines failed to activate NF-κB in the ESC-tenocytes. However, NF-κB could be activated to induce changes in gene expression following stimulation with NF-κB pharmaceutical activators. Transcriptomic analysis revealed differences between cytokine and NF-κB signalling components between adult and ESC-tenocytes, which may contribute to the mechanism by which ESC-tenocytes escape inflammatory stimuli. Further investigation of these molecular mechanisms will help guide novel therapies to reduce fibrosis and encourage superior tendon healing.

**Graphical Abstract:**

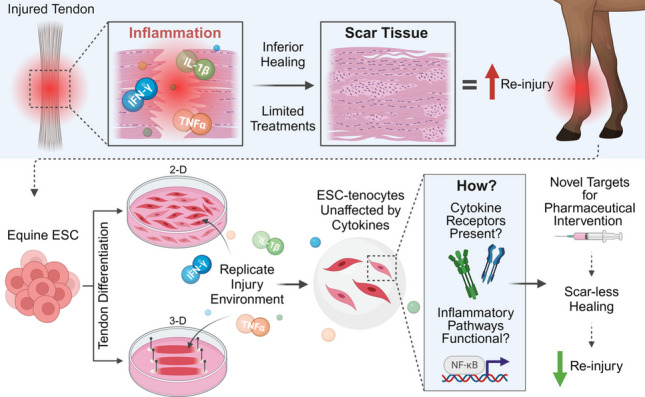

**Supplementary Information:**

The online version contains supplementary material available at 10.1007/s12015-024-10693-8.

## Introduction

The tendon is an essential component of the musculoskeletal system which allows the transmission of contraction forces from skeletal muscle to bone. Certain tendons, such as the equine superficial digital flexor tendon (SDFT) and human Achilles tendon, are highly specialised to facilitate high-speed locomotion and withstand high tensile forces [1, 2]. These properties are a consequence of the tendons' hierarchical structure, comprised of dense, highly organised collagen fibre bundles embedded within an extracellular matrix (ECM) [3]. Tendon injuries are common, accounting for one-third of all musculoskeletal injuries in humans [4] and 46% of all limb injuries in Thoroughbred racehorses [5]. The healing process of an injured tendon is slow, often leading to disorganised tissue involving fibrosis. This can impede the tendon’s ability to fully regain its normal function and structure, leading to reduced strength, flexibility, and elasticity at the lesion site [3, 6], and ultimately making it more susceptible to re-injury (up to 67% in equine athletes [7]). The equine SDFT and the human Achilles tendon share several anatomical and physical characteristics including ageing phenotypes [8] and elastic energy-storing function for efficient locomotion [9, 10]. Thus, the use of equine models for tendinopathy research is a valuable tool in advancing our understanding of tendon injuries and improving the health outcomes of both horses and humans.

The importance of inflammation in both the early initiation of tendon pathologies and the tendon healing process has been recognised in recent years in patient samples from various stages of tendinopathy [11, 12]. Inflammation in tendon injuries is characterised by the endogenous expression of inflammatory mediators including pro-inflammatory cytokines. Amongst these, TNFα (tumour necrosis factor alpha) and IL-1β (interleukin 1 beta) are evidenced to be the most potent inducers of a catabolic response, with recent evidence suggesting these inflammatory cytokines in combination with IFN-γ (interferon gamma) have detrimental consequences for tendon cell function *in vitro* [13]*.* Inflammatory cytokines stimulate the activation of specific signalling pathways inside the cell, including nuclear factor kappa B (NF-κB), mitogen-activated protein kinase (MAPK), c-Jun N-terminal kinase (JNK), and signal transducer and activator of transcription (STATs) [14–16]. Our previous work has demonstrated that in adult tenocytes the adverse effects of TNFα and IL-1β are likely caused by their activation of NF-κB [13, 17]. This triggers a series of inflammatory processes that eventually lead to the transcription of different regulatory genes, linking extracellular signals to the mechanisms that govern essential cellular functions. However, although an inflammatory response is essential to initiate successful wound healing, dysregulation of these signalling pathways may contribute to the degradation of the tendon matrix and ultimately lead to chronic tendinopathy and tissue fibrosis [13, 18–20]. Therefore, understanding how these mechanisms regulate inflammation is critical to identifying therapeutic approaches to promote tendon healing.

Multiple treatment strategies have been recommended for tendinopathy. Although many conservative treatment options resolve the clinical signs associated with a tendon injury, they do not typically result in significant improvements to tendon functionally [21]. More recently, regenerative cell-based strategies which aim to restore tendon tissue with the same biomechanical properties as native tissue have been investigated. Mesenchymal stromal cells derived from bone-marrow aspirates (BM-MSCs) have rapidly advanced to clinical application in horses due to their ability to promote tissue regeneration and reduce fibrosis [22–24]. While BM-MSCs show promise as a regenerative therapy, there are several limitations to their use. These include limited proliferation capacity before entering senescence and the heterogenicity of the cell populations making it difficult to standardise BM-MSC preparation [25]. These limitations highlight a need for the investigation of alternative cell sources for regenerative tendinopathy treatment.

Pluripotent embryonic stem cells (ESCs) are derived from the inner cell mass of blastocyst-stage embryos. These cells offer the potential to advance regenerative treatment strategies, due to their distinguished capacity of unlimited self-renewal and ability to differentiate into derivatives of all three germ layers [26]. In human medicine, the major barriers to the possible transplantation of ESCs into patients are tumour formation and immune rejection [27]. We have previously isolated and characterised equine ESCs [28] and shown they exhibit high survival following injection into an equine SDFT injury, without inducing a cell-mediated immune response or undergoing uncontrolled proliferation during the 90-day study period [29]. Although equine ESCs have currently not been shown to form teratomas when injected into severe combined immunodeficient (SCID) mice [30], studies are yet to investigate the long-term safety and efficacy of these cells.

*In vitro,* equine ESCs undergo tenocyte differentiation in response to transforming growth factor-β3 (TGF-β3) and three-dimensional (3-D) culture in a collagen matrix [31–33]. Transcriptomic profiling of equine ESC-tenocytes has shown that these cells are transcriptomically closer to regenerative fetal tenocytes than adult tenocytes [33]. When stimulated with IL-1β, unlike adult tenocytes, equine ESC-tenocytes exhibit minimal effects on matrix metalloproteinase (*MMP)* or tendon-associated gene expression and generate tendon-like constructs indistinguishable from unstimulated controls [34]. These characteristics may not only have important benefits for their clinical application, but they also allow the use of equine ESC-tenocytes as a model to investigate the key signalling cascades involved in cytokine responses during tendinopathy.

The response of adult tenocytes to pro-inflammatory cytokines has been investigated extensively [13, 17, 34–36], whereas few studies have been performed in equine ESC-derived tenocytes [34]. Here, we determine the responses of equine ESC-tenocytes to IFN-γ, TNFα, and IL-1β. Additionally, the activation of key inflammatory pathways are measured and the mechanisms underpinning ESC-tenocyte responses are investigated.

## Materials and Methods

### Study Design

**Fig. 1:**
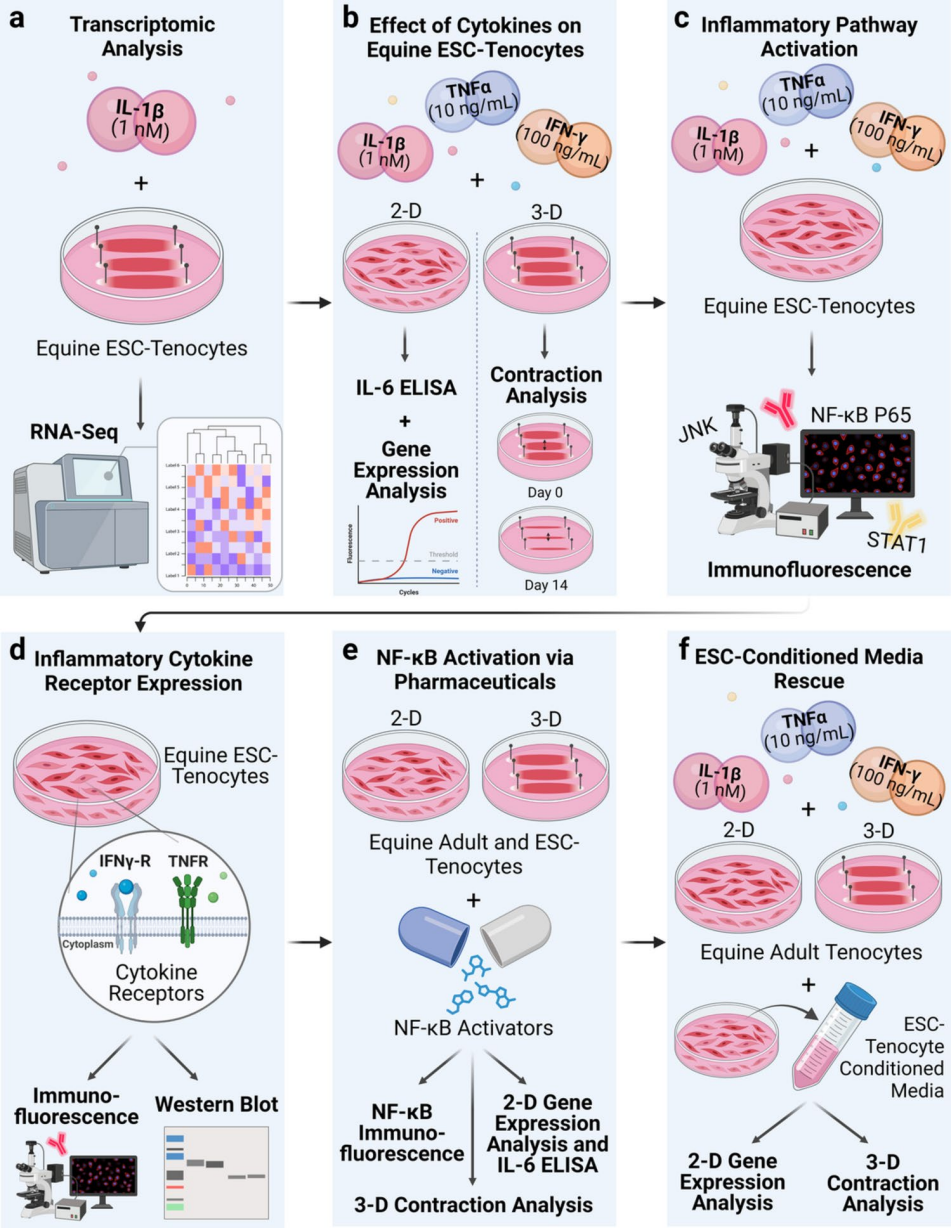
Experimental study design outlining ESC-tenocyte transcriptomic analysis in response to IL-1β (**a**), effect of inflammatory cytokine stimulation on equine ESC-tenocytes (**b**), inflammatory pathway activation (**c**), inflammatory cytokine receptor expression analysis (**d**), activation of NF-κB through pharmaceuticals (**e**), and ESC-tenocyte conditioned media rescue (**f**). Created with BioRender.com

A summary of the study design is shown in Fig. 1. Initially, ESC-tenocytes were cultured in 3-D and stimulated with IL-1β prior to genome-wide transcriptomic analysis (Fig. 1a). Following this, experiments were performed to determine the consequences of IL-1β, TNFα, and IFN-γ exposure on ESC-tenocyte 3-D collagen gel contraction, gene expression in 2-D culture, and IL-6 secretion (Fig. 1b). Activation of key inflammatory signalling pathways (NF-κB P65, STAT1, and JNK) following inflammatory cytokine stimulation was investigated by immunofluorescent cytochemistry (Fig. 1c). Since the inflammatory cytokines had minimal effects on ESC-tenocytes, we examined the expression of the cytokine receptors though immunofluorescence and western blot analysis (Fig. 1d). We next attempted to induce NF-κB P65 activation in ESC-tenocytes through NF-κB pharmaceutical activators, initially determining activation through immunofluorescence, and then studying their effects on gene expression in 2-D culture, IL-6 production, and 3-D collagen gel contraction (Fig. 1e). Finally, we investigated whether conditioned media from ESC-tenocytes possessed the ability to attenuate adverse inflammatory responses by measuring its effects on cytokine-mediated adult tenocyte 2-D gene expression and 3-D collagen gel contraction (Fig. 1f). For all experiments either vehicle only conditions were used as a negative control, or adult tenocytes were used as a positive control since they are known to be adversely affected by inflammatory cytokine stimulation [13].

### Tenocyte Cell Isolation and Culture

Adult tenocytes were isolated post-mortem from sixteen Thoroughbred type horses (aged between two and thirteen years old) that had been euthanized for reasons unrelated to this research with approval from the Royal Veterinary College Clinical Research Ethical Review Board (URN 2020 2017–2). As described previously [32], dissected SDFT tissue was incubated with 1 mg/mL type I collagenase from *Clostridium histolyticum* (Sigma-Aldrich, Dorset, UK) overnight at 37 °C. The resulting cells were expanded and cultured in growth media consisting of Dulbecco’s modified Eagle’s medium (DMEM; high glucose [4500 mg/L] with sodium pyruvate [110 mg/L]) supplemented with 10% fetal bovine serum (FBS), 1% penicillin–streptomycin (P/S) and 2 mM L-glutamine (LQ) (all Gibco, Thermo Fisher, Hemel Hempstead, UK). Culture conditions were maintained at 37 °C in a humidified atmosphere of 5% CO_2_. Tenocytes were passaged enzymatically using 0.25% trypsin–EDTA (Sigma-Aldrich) every 3–4 days prior to reaching maximal confluency.

### Embryonic Stem Cell Culture and Tenocyte Differentiation

Previously characterised equine ESCs from three different embryos were used in this research [28, 29, 31, 32, 37]. ESCs were cultured at 37 °C in a humidified atmosphere of 5% CO_2_ on mitotically inactivated mouse embryonic fibroblasts (MEFs) in ESC growth medium (DMEM/F12 supplemented with 15% FBS, 2 mM LQ, 1% non-essential amino acids, 1 mM sodium pyruvate, and 0.1 mM 2-mercaptoethanol; all Gibco, Thermo Fisher) and 1000 units/mL recombinant human leukaemia inhibitory factor (LIF) (Peprotech, London, UK) to maintain pluripotency. ESC growth medium was replenished daily, and cells were passaged mechanically every 6–8 days in the presence of 2 μM Thiazovivin (Miltenyi Biotech, Surrey, UK). For 2-D tenocyte differentiation, ESCs were cultured for 14 days without MEFs in ESC growth media without LIF, but with 20 ng/mL transforming growth factor beta-3 (TGF-β3) (Peprotech). Conditioned media from ESC-Tenocytes was obtained by removing the ESC growth media supplemented with TGF-β3 at day 14 and replenishing it with fresh ESC growth media lacking LIF and TGF-β3. This “ESC-tenocyte-conditioned media” was collected after 48 h of culture and centrifuged (130 × *g* for 3 min) to remove cell debris before being used immediately or stored at -70 °C until needed.

### Three-Dimensional Cell Culture of Tenocytes

Three-dimensional (3-D) culture of tenocytes was performed as previously described [13, 31, 34]. Briefly, pairs of 0.2-mm-diameter minutien pins (InterFocus, Cambridge, UK) were embedded in silicon-coated six-well culture plates (Sylgard 184 Silicone elastomer; Farnell, Leeds, UK) 15 mm apart. Adult tenocytes (4 × 10^5^ cells/mL) were suspended in a chilled mixture of 2 parts growth medium and 8 parts PureCol (Bovine collagen type I; Advanced Biomatrix, Carlsbad, USA). The pH of the resulting collagen-cell suspension was adjusted to 7.2–7.6 with 1 M sodium hydroxide and immediately pipetted (200 μL) between each pair of minutien pins. The plate with attached lid was then sealed with parafilm and incubated at 37 °C for 60–90 min. Once set, the 3-D tendon constructs were cultured in growth media alone (unstimulated control) or supplemented with recombinant human TNFα (10 ng/mL), recombinant human IL-1β (17 ng/mL), and/or recombinant equine IFN-γ (100 ng/mL; all PeproTech) [13], or NF-κB activators Prostratin (2 μM) and Phorbol 12-myristate 13-acetate (PMA) (10 ng/mL; both Abcam, Cambridge, UK). For ESC seeded constructs, mechanically passaged pieces of undifferentiated ESCs were used to prepare the 3-D tendon constructs at the same cell density (representative cell counts performed using cells detached with TrypLE™ Select; Invitrogen) and were cultured in ESC growth medium without LIF [31]. All cultures were maintained for 14 days at 37 °C in a humidified atmosphere of 5% CO_2_, and media was replaced every 3–4 days during this time. For each of these experiments, three to five biological replicates of adult tenocytes (P1 – P7) and three biological replicates of ESC-tenocytes (P12 – P34) were used.

ImageJ software (National Institutes of Health, USA) was used to analyse the contraction rate of the 3-D constructs over time with values displayed as a percentage of the day 0 value. At day 14, cell survival was determined by digesting the constructs in 1 mL of growth media with 1 mg/mL type I collagenase from *Clostridium histolyticum* (Sigma-Aldrich) for 20 min – 1 h at 37 °C. Cell survival was calculated as a percentage of the seeded cell number on day 0.

### RNA Isolation and Extraction

For 2-D stimulation experiments, ESC-tenocytes were stimulated with TNFα (10 ng/mL), IL-1β (17 ng/mL) and IFN-γ (100 ng/mL) alone or in combination for 72 h prior to harvesting the cells for RNA. For NF-κB activator pharmaceutical experiments, adult and ESC-tenocytes were stimulated with Prostratin (2 μM) or PMA (10 ng/mL) for 72 h before harvesting the cells for RNA. RNA was extracted using Tri-reagent (Sigma-Aldrich) followed by purification using an RNeasy mini kit (Qiagen, Manchester, UK). Contaminating genomic DNA was removed using a DNA-free™ DNA removal kit (Invitrogen, Thermo Fisher) and RNA concentration and purity were measured using a DeNovix DS-11 Spectrophotometer (DeNovix, Wilmington, USA).

### RNA Sequencing

RNA sequencing was previously performed using five biological replicates of adult tenocytes (between P3 and P6) cultured in 3-D in the presence or absence of IL-1β (17 ng/mL) (sequence data available via the National Centre for Biotechnology Information Gene Expression Omnibus accession number GSE221370) [17]. Here, we performed RNA sequencing on three biological replicates of ESC-tenocytes (between P10 and P32) cultured in 3-D in the presence or absence of IL-1β (17 ng/mL). RNA was isolated as described above, concentration was determined using a Qubit (Thermo Fisher) and samples were confirmed to have a 260:280 ratio between 1.8 and 2.2 using a DeNovix DS-11 Spectrophotometer. RNA integrity values of over 8 were confirmed using a Tapestation (Agilent, Milton Keynes, UK). As previously described [17], mRNA libraries were prepared from total RNA and converted to end-paired cDNA with adapter ligation by an external provider (Novagene, Cambridge, UK). Libraries were size selected, multiplexed, and quality controlled before RNA sequencing was performed on a NovaSeq6000 platform, generating 30.7–38 million reads of 150 base pair paired-end data per sample. FASTQC (version 0.11.9; Babraham Bioinformatics, Cambridge, UK) was used to quality control check the resulting FASTQ files. Quality checked reads were aligned to the Equus caballus transcriptome (EquCab3.0 GCF_002863925.1) using the pseudoaligner Salmon [38] (version 1.8) in Quasi-mapping-based mode with GC-bias correction (-gcBias). Quantified gene level abundance data was imported into R (version 4.2.1) using Tximport [39] (version 1.24). Differential expression analysis was performed using R/Bioconductor DESeq2 software (version 1.36). Genes with a log2-fold change (Log2FC) of ± 1 and an adjusted *p* < 0.05 were considered differentially expressed (DE).

### cDNA Synthesis and Quantitative PCR

RNA (500 ng) was reverse transcribed using the SensiFAST™ cDNA Synthesis Kit (Bioline, London, UK). To ensure there was no genomic DNA contamination, reactions lacking the reverse transcriptase were also performed. Equine compatible gene primers were designed using primer3 (http://primer3.ut.ee) and NCBI Primer Blast (https://www.ncbi.nlm.nih.gov/tools/primer-blast/) ensuring each amplicon had a melting temperature (Tm) between 58–62 °C, lacked a secondary structure at Tm 60 °C, and had an amplicon size of 50–150 bp. Primer sequences are shown in Table 1 qPCR reactions were performed in duplicate using 20 ng of cDNA with a SYBR Green containing supermix (Bioline) on a C1000 Touch Thermal Cycler (Bio-Rad, Hertfordshire, UK). qPCR cycling parameters were 95 °C (10 min), followed by 45 cycles of 95 °C (15 s), 60 °C (15 s) and 72 °C (15 s). Subsequently, a melt curve was produced with readings taken every 1 °C from 65 to 95 °C. Relative gene expression levels were normalised with the housekeeping gene *18 s rRNA* using the 2^−ΔΔCt^ method [13, 40]. Adult tenocytes from three or four biological replicates (P3—P8) and ESC-tenocytes from three biological replicates (P13—P25) were used in these experiments.

**Table 1:** Primer sequences used for qPCR analysis

Gene	Protein Name	Gene ID	Forward Primer	Reverse Primer
*18 s rRNA*	18 s ribosomal RNA	100,861,557	CCCAGTGAGAATGCCCTCTA	TGGCTGAGCAAGGTGTTATG
*SCX*	Basic helix-loop-helix transcription factor scleraxis	100,125,857	CCCAAACAGATCTGCACCTT	ATCCGCCTCTAACTCCGAAT
*TNMD*	Tenomodulin	100,033,840	GTCCCTCAAGTGAAGGTGGA	CCTCGACGGCAGTAAATACAA
*TNC*	Tenascin	100,049,835	AACCCGTCCAAAGAGACCTT	GCGTGGGATGGAAGTATCAT
*COL1A1*	Collagen type I alpha 1 chain	100,033,877	TGCGAAGACACCAAGAACTG	GACTCCTGTGGTTTGGTCGT
*COL5A1*	Collagen type V alpha 1 chain	100,069,057	AGGAGAGAGAGGCCCAAAAG	CTCCATCAATTCCCTGAG GA
*COMP*	Cartilage oligomeric matrix protein	100,033,911	AGAACATCATCTGGGCCAAC	CGCTGGATCTCGTAGTCCTC
*THBS4*	Thrombospondin-4	100,073,261	GGGAAATGGGGTTACCTGTT	CGGGTAGCAGGGATGATATT
*SOX9*	Transcription factor SOX-9	100,033,908	GCTCTGGAGACTTCTGAACGA	GTAATCCGGGTGGTCCTTCT
*IL-6*	Interleukin 6	100,034,196	GAAAGAGAGCTTCATCTGCCC	ACTGGAGTGACGGTGCTTG
*MKX*	Homeobox protein Mohawk	100,065,010	AAGGCAAAGGAACCATTCGGTTA	TTAGCTGTCACCCTTATTGGAT
*MMP1*	Matrix Metallopeptidase 1 (Interstitial collagenase)	100,033,896	CTTTGATGGACCTGGAGGAA	GAATGGCCAAATTCATGAGC
*MMP2*	Matrix Metallopeptidase 2 (72 kDa type IV collagenase)	100,033,948	CAGGAGGAGAAGGCTGTGTT	AGGGTGCTGGCTGAGTAGAC
*MMP3*	Matrix Metallopeptidase 3 (Stromelysin-1)	100,034,195	TGGACCTGGAAAAGTTTTGG	GACCAAGTTCATGAGCAGCA
*MMP8*	Matrix Metallopeptidase 8 (Neutrophil collagenase)	100,069,005	TTTGATGGACCCAATGGAAT	TTCATGGGCAGCAACAATAA
*MMP9*	Matrix Metallopeptidase 9	100,056,599	GAGATCGGGAATCATCTCCA	CCAAGAGTCGCCAGTACCTC
*MMP13*	Matrix Metallopeptidase 13 (Collagenase 3)	100,009,711	GCCACTTTGTGCTTCCTGAT	CGCATTTGTCTGGTGTTTTG

### MMP Activity Assay

Total MMP activity was determined using the MMP activity assay kit (ab112146, Abcam) according to the manufacturer’s specifications. Media was harvested from four biological replicates of adult tenocytes (P4 – P6) and three biological replicates of ESC-tenocytes (P14 – P20) cultured for 72 h with growth media alone (unstimulated control) or growth media containing TNFα (10 ng/mL), IL-1β (17 ng/mL), and IFN-γ (100 ng/mL) and stored at -70 °C until use. For the MMP assay, the media was first activated with an equal volume of 2 mM APMA working solution and incubated at 37 °C for 2 h. Each sample was then mixed with MMP Green Substrate and incubated for 1 h at room temperature prior to performing an endpoint fluorescent reading at Ex/Em = 490/525 nm on a Tecan plate reader (Infinite M Plex; Tecan, Switzerland). The baseline was determined with a negative substrate control and subtracted from the individual samples to determine the measured activity of the MMPs.

### Presto Blue Cell Viability Assay

Cell viability of three biological replicates of adult tenocytes (P4 – P8) was measured using a PrestoBlue™ cell viability reagent (Invitrogen, Thermo Fisher). Adult tenocytes were seeded onto 96-well tissue culture plates at 1.5 × 10^4^ cells per well in growth media. Cells were incubated at 37 °C and 5% CO_2_ for 24 h to allow for cell adhesion. After 24 h, fresh growth media alone (unstimulated control) or with NF-κB activators Prostratin (2—50 μM) or PMA (10—100 ng/mL) (both Abcam) was added. After 72 h incubation, growth media was removed from the cells and 100 μL of diluted PrestoBlue™ reagent (1:10) was added to each well and the plates incubated at 37 °C for 30 min. Fluorescence was measured at an excitation wavelength of 560 nm and an emission of 590 nm on a Tecan plate reader (Infinite M Plex; Tecan).

### Immunofluorescence

For each immunofluorescence experiment, three biological replicates of adult tenocytes were used (between P2 – P7). Initially, adult tenocytes were cultured on gelatin-coated (Sigma-Aldrich) coverslips in 24-well tissue culture plates. Once the cells had reached 70–80% confluency, they were stimulated with NF-κB activators Prostratin (0.2, 2, 10 μM), PMA (10, 50, 100 ng/mL), Betulinic acid (1, 10, 100 μg/mL), or A23187 (0.2, 2, 10 μM) for various timepoints between 30 min and 24 h to establish optimal treatment conditions. Once established, three biological replicates of equine ESCs (between P9 – P33) were differentiated into tenocytes directly on gelatin-coated coverslips and subsequently treated with TNFα (10 ng/mL), IL-1β (17 ng/mL), and/or IFN-γ (100 ng/mL) for 1 h or NF-κB activators Prostratin (2 μM, 30 min), PMA (10 ng/mL, 1 h), Betulinic acid (10 μg/mL, 1 h), or A23187 (2 μM, 1 h). Cells cultured with growth media alone served as controls. Following stimulation, cells were fixed in 3% paraformaldehyde for 20 min, permeabilised with 0.1% Triton-X-100 (Sigma-Aldrich) for 1 h at room temperature and then blocked with 2.5% normal horse serum (NHS) (Vector Laboratories, Peterborough, UK) for 20 min also at room temperature. Incubation with the primary antibody (see Table 2) was carried out in 2.5% NHS overnight in a humidified atmosphere at 4 °C. Secondary antibody incubation was performed using either goat anti-mouse IgG Alexa Fluor 594 (1:200) or goat anti-rabbit IgG Alexa Fluor 594 (1:200; both Thermo Fisher) in 2.5% NHS for 3 h at room temperature. The use of the secondary antibody only served as a negative control. Coverslips were mounted using Vectashield Hardset with DAPI (4′,6-diamidino-2-phenylindole; Vector Laboratories). Fluorescent images were taken using a Nikon Eclipse Ti2 series microscope (Nikon, Surrey, UK). Nuclear fluorescent intensity was quantified by measuring the mean grey scale of the nuclear region using ImageJ software.

**Table 2:** Primary antibodies used for immunofluorescence

Marker	Species	Clone	Dilution	Company	References
NF-κB (P65)	Mouse	Monoclonal 572	1:100	Thermo Fisher (436700)	[13]
STAT1	Rabbit	Monoclonal EPR4407	1:200	Abcam, UK (ab109320)	[13]
JNK 1,2,3	Rabbit	Monoclonal EPR16797-211	1:200	Abcam (ab179461)	[13]
TNFR1	Rabbit	Polyclonal	1:200	Biorbyt, Cambridge, UK (orb107276)	Cross-reactivity shown by western blot (Fig. 5)
TNFR2	Rabbit	Monoclonal EPR1653	1:100	Abcam (ab109322)	Cross-reactivity shown by western blot (Fig. 5)
IFNGR1	Rabbit	Polyclonal	1:200	Assay Biotechnology, Fremont, CA (B0953)	Cross-reactivity shown by western blot (Supplementary Fig. 4)
IFNGR2	Rabbit	Polyclonal	1:100	Biorbyt (orb415521)	Cross-reactivity shown by western blot (Supplementary Fig. 4)

### Western Blot Analysis

Cytoplasmic protein fractions were isolated from three biological replicates of adult (P4—P7) and ESC-tenocytes (P15—P26) using the NE-PER™ Nuclear and Cytoplasmic Extraction Reagents and Halt™ Protease Inhibitor Cocktail (100X; both Thermo Fisher) according to the manufacturer’s instructions. Fifteen to thirty micrograms of reduced cytoplasmic protein were loaded into each well of a NuPAGE 4–12% Bis–Tris Gel (Invitrogen). Following transfer, the nitrocellulose membrane (Cytiva™, Buckinghamshire, UK) was probed with antibodies for TNFR1 (1:1000), TNFR2 (1:1000), IFNGR1 (1:1000) and IFNGR2 (1:1000). Immunoreactivity was detected using goat anti-rabbit IgG H&L (HRP) (Abcam, ab6721, 1:5000–1:7000) or goat anti-mouse IgG H&L (HRP) (Abcam, ab6789, 1:10,000) and the ECL plus Western Blotting Substrate (Pierce™, Thermo Fisher) on a G:BOX (Syngene, Cambridge, UK). The mouse anti-β actin antibody (Abcam, ab8226, 1:1000) was used as a positive control. Densitometric quantification of Western blots was performed using Image Lab 6.1 software (Bio-rad). The measurements were normalised to total protein stain (Ponceau S; Sigma-Aldrich) [41].

### IL-6 and IL-1RA ELISA

Commercially available equine ELISA kits (R&D systems, Abingdon, UK) were used to determine the concentrations of IL-6 and IL-1RA in 72 h conditioned media samples according to the manufacturer’s instructions. Colorimetric detection was performed on a Tecan plate reader (Infinite M Plex) at 450 nm with background correction at 540 nm. A seven-point standard curve with four-parameter logistic regression was used to determine the concentration of IL-6 (pg/mL) or IL-1RA (ng/mL). Adult tenocytes from three biological replicates (P2 – P8) and ESC-tenocytes from three biological replicates (P9—P31) were used in these experiments.

### ESC-Tenocyte Conditioned Media and Tenocyte Co-Culture

ESC-tenocyte conditioned media experiments were performed using three biological replicates of ESC-tenocytes (P9 – P30) and three or four biological replicates of adult tenocytes (P1—P6). Adult tenocytes were cultured in 2-D or 3-D as described previously in either i) ESC growth media, ii) ESC growth media containing cytokines (TNFα (10 ng/mL), IL-1β (17 ng/mL), and IFN-γ (100 ng/mL)), iii) previously prepared ESC-tenocyte conditioned media alone (see above), or iv) ESC-tenocyte conditioned media containing cytokines (TNFα (10 ng/mL), IL-1β (17 ng/mL), and IFN-γ (100 ng/mL)). 2-D cultures were harvested for RNA after 72 h and qPCR analysis performed. 3-D cultures of adult tenocytes were maintained for 14 days with media changes every 3–4 days.

### Statistical Analysis

All statistical analysis was performed using SPSS (version 28.0.0.0.; IBM, UK). Gaussian distribution of all data was tested using the Shapiro-Wilks normality test and equal variance using Levene’s test. For comparisons of two groups, independent Students t-tests (unpaired, two-tailed) were used. For comparisons of more than two groups, a one-way ANOVA with *post-hoc* Tukey was used. Where data was not normally distributed, it was log-transformed prior to analysis. If normality was still not met a non-parametric Kruskal Wallis test was performed followed by Dunn’s pairwise comparisons. If data was normally distributed but with unequal variance, Welch’s ANOVA was used followed by Games-Howell *post hoc*. For comparisons of more than two groups over time, a two-way ANOVA with a Bonferroni *post hoc* correction for multiple comparisons was used. If the assumption of sphericity was violated, a greenhouse–geisser correction was applied. In all cases, a *p* value of < 0.05 was considered statistically significant.

## Results

### IL-1β Stimulation Fails to Elicit Changes in Global Gene Expression Patterns by ESC-Tenocytes After 14 Days in 3-D Culture

**Fig. 2:**
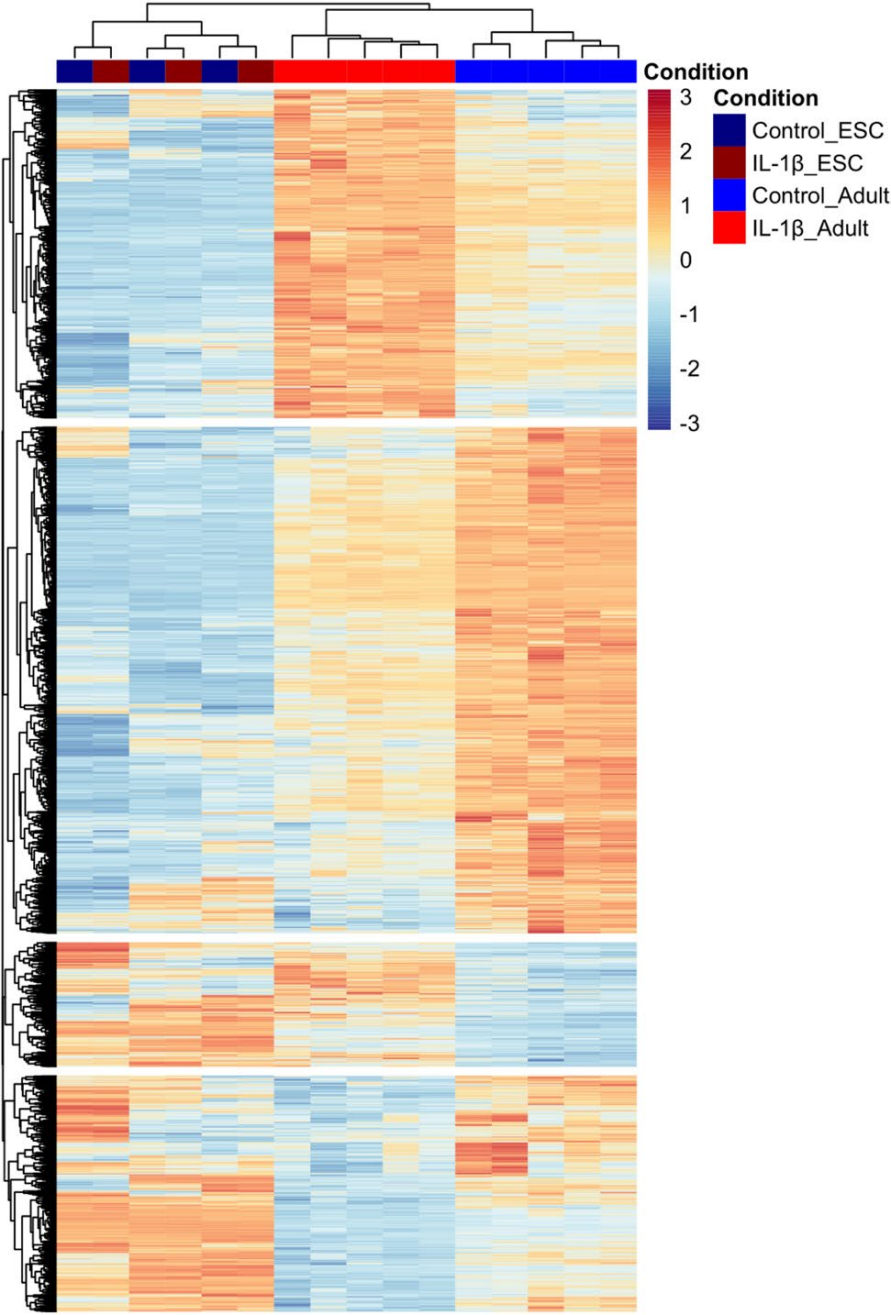
Transcriptomic responses of equine adult (Beaumont et al*.*, 2023, GSE221370) and ESC-derived tenocytes stimulated with IL-1β for 14 days in 3-D culture. The heatmap shows the normalised counts for the 2517 DE genes in adult tenocytes [17], relative to the 0 DE genes in ESC-tenocytes (adjusted *p* value < 0.05 and log2-fold change ± 1). Samples are visualised in columns (control in blue, IL-1β in red) and genes are represented and clustered (Euclidean) by row. For this comparison, five biological replicates of adult tenocytes (between P3 and P6) and three biological replicates of ESC-tenocytes (between P10 and P32) were used

Previously, IL-1β demonstrated adverse effects on tendon-associated gene expression in adult tenocytes in 2-D culture, however, ESC-tenocytes appeared to be unaffected by IL-1β stimulation [34]. In 3-D culture, transcriptomic analysis revealed exogenous IL-1β simulation of adult tenocytes results in 2517 differentially expressed (DE) genes, with 954 and 1653 up and down-regulated in expression, respectively [17]. Here, we evidence that ESC-tenocytes exhibited no DE genes following IL-1β stimulation in 3-D culture (Fig. 2 and Supplementary Fig. 1a and b). qPCR analysis of selected genes validated this finding (Supplementary Fig. 1c).

### IL-1β, TNFα, and IFN-γ Stimulation Results in Minimal Changes to 2-D Tendon-Associated Gene Expression or 3-D Collagen Gel Contraction by ESC-Tenocytes

**Fig. 3:**
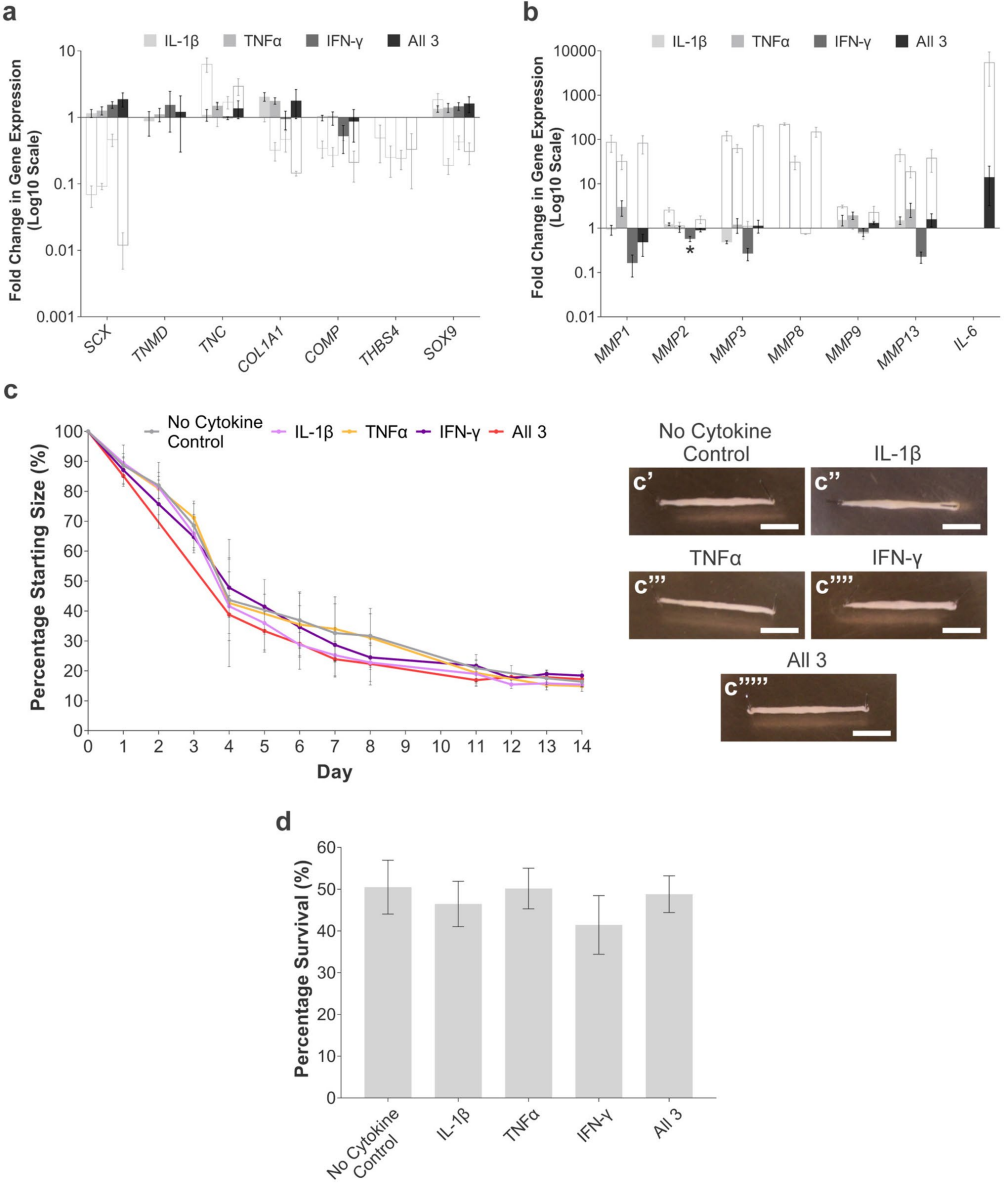
The effect of inflammatory stimulation on 2-D gene expression and 3-D collagen gel contraction by ESC-tenocytes. Fold change in tendon-associated (**a**) and *MMP* (**b**) gene expression in ESC-tenocytes following stimulation with IFN-γ, TNFα, and/or IL-1β compared to the no cytokine control. Open bars represent existing data from adult tenocytes [13] to act as a comparison. *IL-6* expression was analysed for ‘All 3’ cytokine treated samples only. (**c**) Inflammatory cytokines either alone or in combination had no effect on the degree of 3-D collagen gel contraction by ESC-tenocytes. Representative images at day 14 are shown in c’-c’’’’’. Scale bar = 5 mm. (**d**) No significant differences in cell survival were found between cytokine treated and no cytokine control gels. The asterisk (*) indicates significant difference relative to no cytokine control *p* < 0.05. Error bars represent the S.E.M of three biological replicates. Cells in these experiments were between P13 – P36

A combination of IL-1β, TNFα, and IFN-γ can produce greater detrimental consequences on adult tenocytes than individual cytokine stimulation [13]. We, therefore, performed qPCR analysis to determine if there are changes in tendon-associated gene expression in ESC-tenocytes cultured in 2-D following 72 h stimulation with a combination of inflammatory cytokines. Stimulation of ESC-tenocytes with IL-1β, TNFα, or IFN-γ alone, or in combination, resulted in minimal changes to both tendon-associated, *IL-6,* and *MMP* gene expression (Fig. 3a and b). This contrasted with our previous work in adult tenocytes (displayed as open bars in Fig. 3a and b) [13]. The qPCR data was confirmed at the protein level by ELISA. As shown in supplementary Fig. 2, cytokine stimulation significantly increased IL-6 secretion and total MMP activity in adult tenocytes, but not in ESC-tenocytes. In 3-D culture, IL-1β, TNFα, or IFN-γ alone or in combination exhibited no significant effect on the final degree of collagen gel contraction by ESC-tenocytes (Fig. 3c) or ESC-tenocyte survival (Fig. 3d) compared to the unstimulated control.

### Inflammatory Pathway Activation in Response to IL-1β, TNFα, and IFN-γ Stimulation in ESC-Tenocytes

**Fig. 4:**
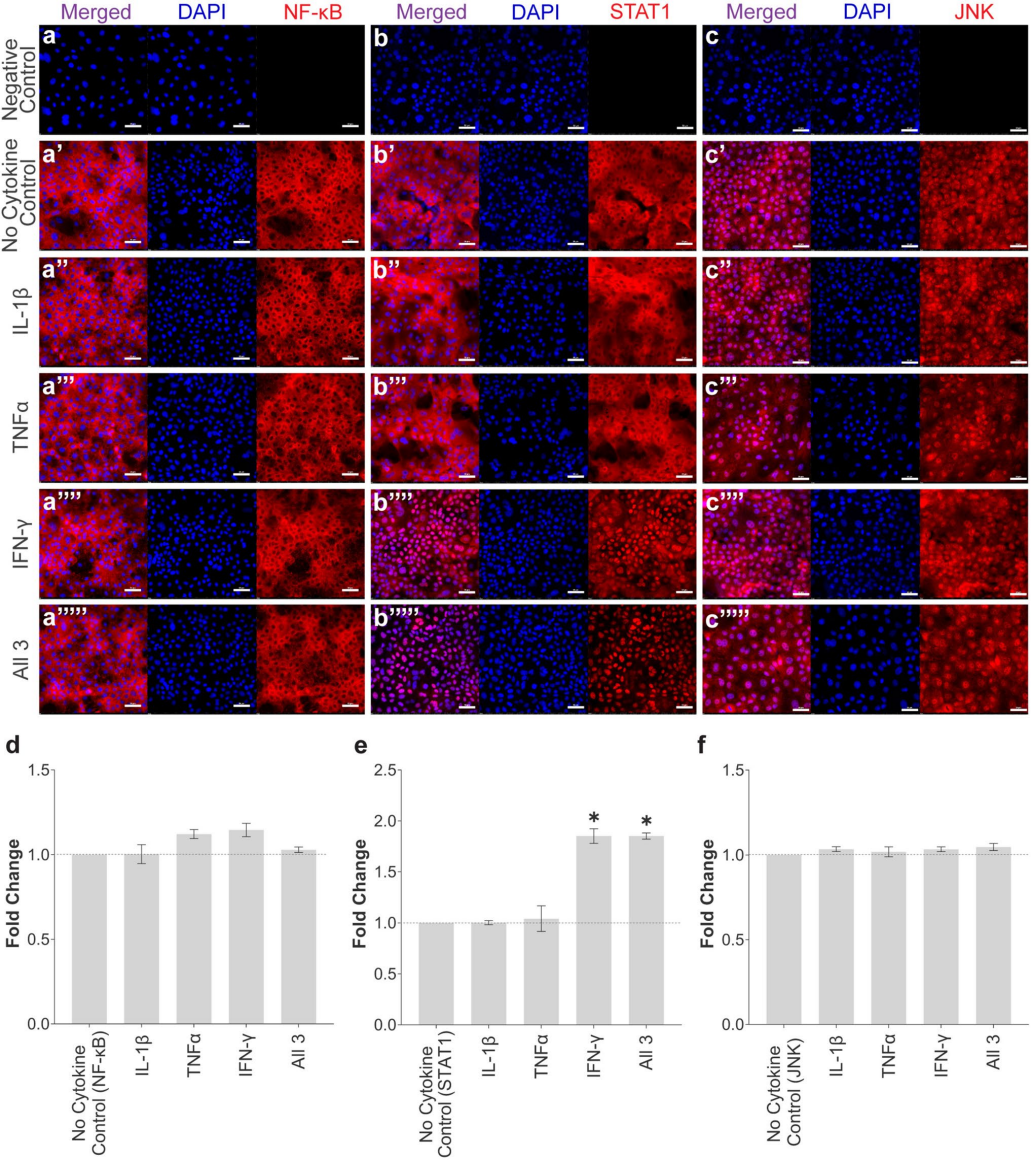
The localisation and activation of inflammatory pathway proteins in response to inflammatory cytokine stimulation. Immunofluorescence staining of NF-κB P65 (a-a’’’’’), STAT1 (b-b’’’’’), and JNK (c–c’’’’’) in ESC-tenocytes following stimulation with IFN-γ, TNFα, and/or IL-1β. Unstimulated cells served as a control. Nuclear staining is shown by DAPI in blue. All images are representative of three independent biological replicates. Scale bar = 50 μm. Semi-quantitative analysis of the relative nuclear fluorescent intensity of NF-κB P65 (d), STAT1 (e), and JNK (f) following inflammatory cytokine stimulation is also shown. Data is displayed as fold change compared to the no cytokine control. Error bars depict the S.E.M of three measurements from three independent biological replicates of ESC-tenocytes. The asterisk (*) denotes the fold change is significantly different to the no cytokine control at *p* < 0.05. Cells in these experiments were between P9 and P21

Immunofluorescent staining demonstrated that IL-1β, TNFα, and/or IFN-γ failed to induce NF-κB P65 nuclear translocation in ESC-tenocytes following 1 h of stimulation (Fig. 4a and d). In contrast, immunofluorescence revealed that exposure to IFN-γ (either alone or in combination with IL-1β and TNFα) significantly induced STAT1 activation compared to the no cytokine control (Fig. 4b and e). Furthermore, basal nuclear JNK localisation in ESC-tenocytes (Fig. 4c) was not significantly affected by any cytokine condition (Fig. 4f).

### Transcriptomic Analysis Demonstrates Differences Between the Expression of NF-κB Components and Cytokine Receptors Between Adult and ESC-Tenocytes; but have Comparable Protein Levels of the TNFα and IFN-γ Receptors

**Fig. 5:**
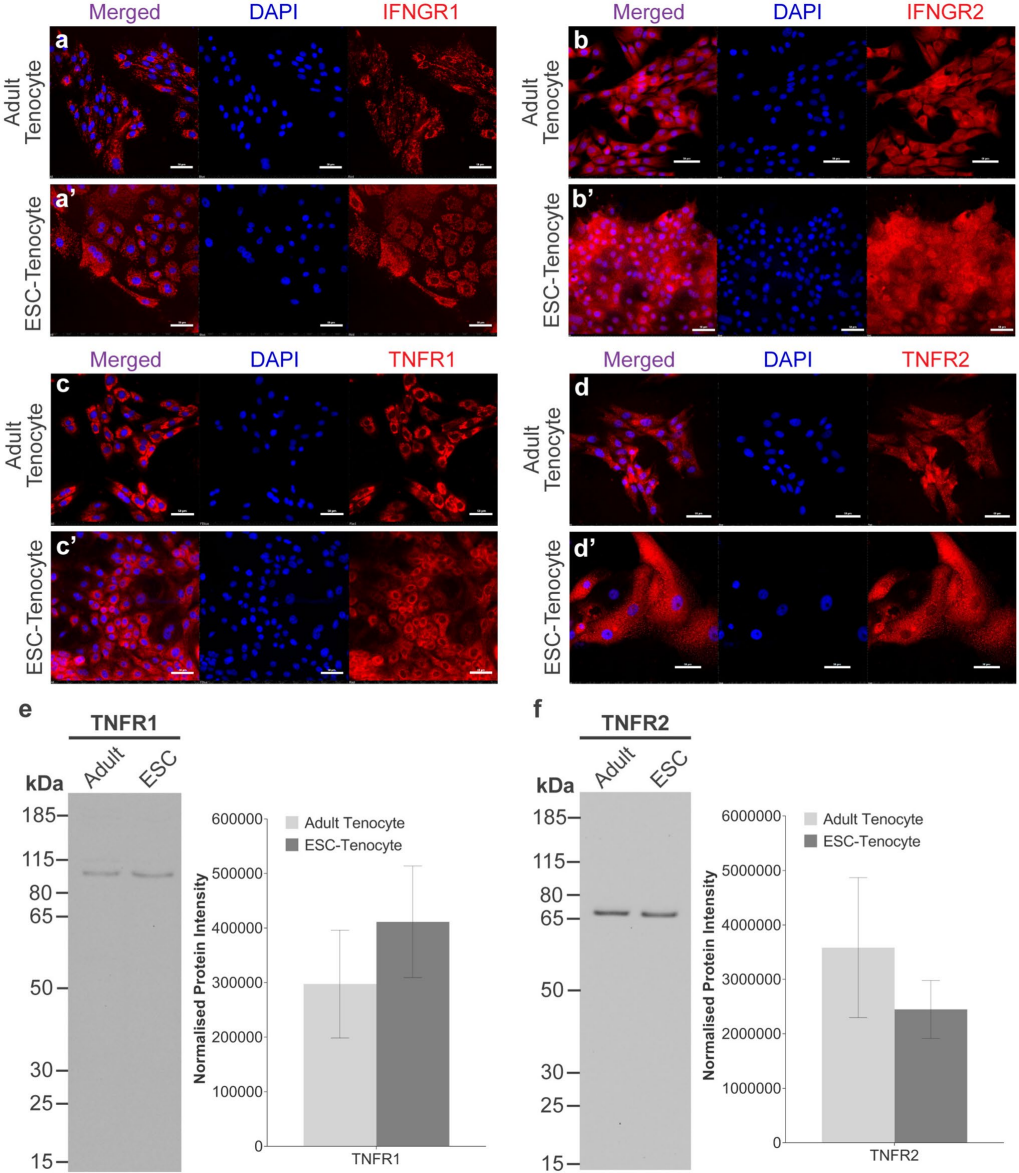
Adult and ESC-tenocytes express comparable protein levels of TNFα and IFN-γ receptors. Immunofluorescence staining of IFNGR1 (**a-a**’), IFNGR2 (**b-b**’), TNFR1 (**c–c**’), and TNFR2 (**d-d**’) in adult and ESC-tenocytes. Nuclear staining is shown by DAPI in blue. All images are representative of three independent biological replicates. Scale bar = 50 μm. Western blot analyses comparing the expression of TNFR1 (**e**) and TNFR2 (**f**) between adult and ESC-tenocytes. Quantitative analyses of normalised protein intensity are also shown. Error bars represent the S.E.M of three biological replicates. In these experiments adult tenocytes were between P2—P7 and ESC-tenocytes between P15—P26

Transcriptomic profiles of adult and ESC-tenocytes from publicly available data (Paterson et al*.*, 2020, GSE145029) were used to compare the expression of key NF-κB pathway and TNF/IL-1 signalling components (Supplementary Fig. 3). Despite adult tenocytes consistently expressing higher levels of several NF-κB signalling components compared to the ESC-tenocytes, this was not significant for any gene examined. In contrast, ESC-tenocytes expressed significantly higher levels of *NFKBIA, NFKBIE,* and *IKBKB* compared to the adult tenocytes. When comparing the expression levels of cytokine receptors, adult tenocytes expressed significantly more *TNFRSF1A* and *IL-1R1*, whereas ESC-tenocytes expressed significantly more *IL-1R2* and *IL-1RN.* Previously, we demonstrated at the protein level that ESC-tenocytes lack expression of IL-1 receptor 1 and express higher levels of the decoy receptor IL-1 receptor 2 compared to adult tenocytes [34]. The induction of STAT1 following exposure to IFN-γ and our previous work demonstrating an increase in MHC-1 expression following IFN-γ treatment suggests that the IFN-γ receptors are present in ESC-tenocytes [36]. Here, we confirm that both adult and ESC-tenocytes express IFNGR1 and IFNGR2 (Fig. 5a and b). Western blot analysis confirmed that these antibodies specifically recognise equine proteins (Supplementary Fig. 4). Additionally, we demonstrate that despite not responding to TNFα, at the protein level the ESC-tenocytes express both TNFR1 and TNFR2 (Fig. 5c and d) at comparable levels to the adult tenocytes (Fig. 5e and f).

### NF-κB P65 can be Activated by Pharmaceuticals in Both Adult and ESC-Tenocytes

**Fig. 6:**
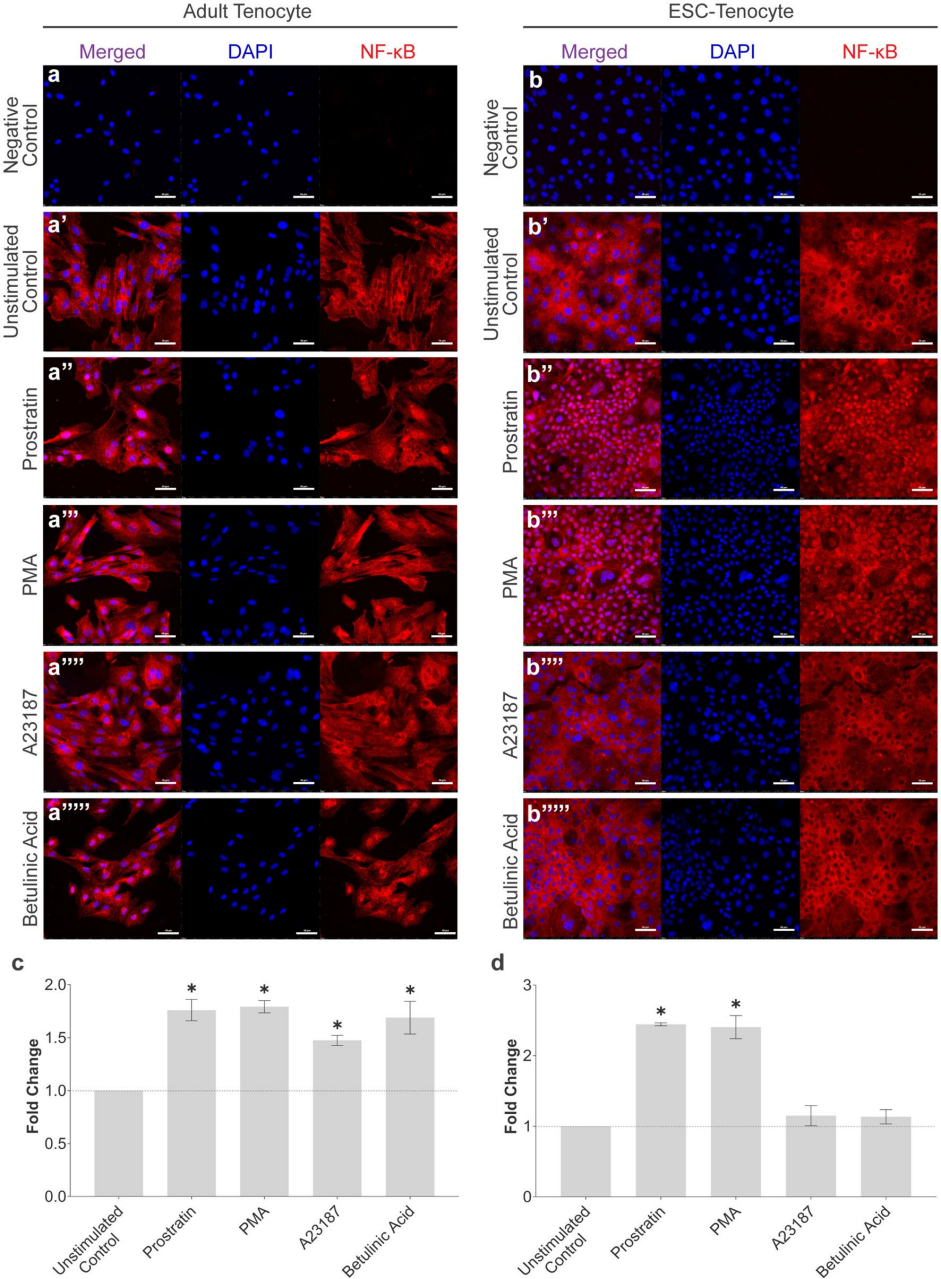
NF-κB signalling can be activated in ESC-tenocytes through NF-κB activator pharmaceuticals. Immunofluorescence staining of NF-κB P65 in adult (a-a’’’’’) and ESC-tenocytes (b-b’’’’’) following 30 min of stimulation with Prostratin (a’’ and b’’) and 1 h stimulation with PMA (a’’’ and b’’’), A23187 (a’’’’ and b’’’’), or Betulinic Acid (a’’’’’ and b’’’’’). DAPI staining of the nucleus is shown in blue. Images are representative of three biological replicates. Scale bar = 50 μm. Semi-quantitative analysis of the relative nuclear fluorescent intensity of NF-κB P65 following NF-κB activator stimulation in adult (c) and ESC-tenocytes (d) is shown. Data is displayed as fold change compared to the unstimulated control. Error bars represent the S.E.M of three measurements from three independent biological replicates. The asterisk (*) denotes the fold change is significantly different to the unstimulated at *p* < 0.05. Adult tenocytes in these experiments were between P3 and P6 and ESC-tenocytes between P9 and P33

Since the NF-κB P65 pathway was not activated by inflammatory cytokines in ESC-tenocytes, we sought to determine whether this pathway was functional using NF-κB pharmaceutical activators. Initially, dose and time response experiments were carried out in adult tenocytes to determine the optimal conditions to induce P65 nuclear translocation (data not shown). Immunofluorescence demonstrated that all four NF-κB pharmaceutical activators induced significant P65 nuclear translocation compared to the unstimulated control within 1 h of stimulation in adult tenocytes (Fig. 6a and c). However, only two (Prostratin and PMA) resulted in significant P65 nuclear translocation in ESC-tenocytes (Fig. 6b and d).

### NF-κB Activators Modulate Tendon-Associated Gene Expression but do not Affect 3-D Collagen Gel Contraction by ESC-Tenocytes

**Fig. 7:**
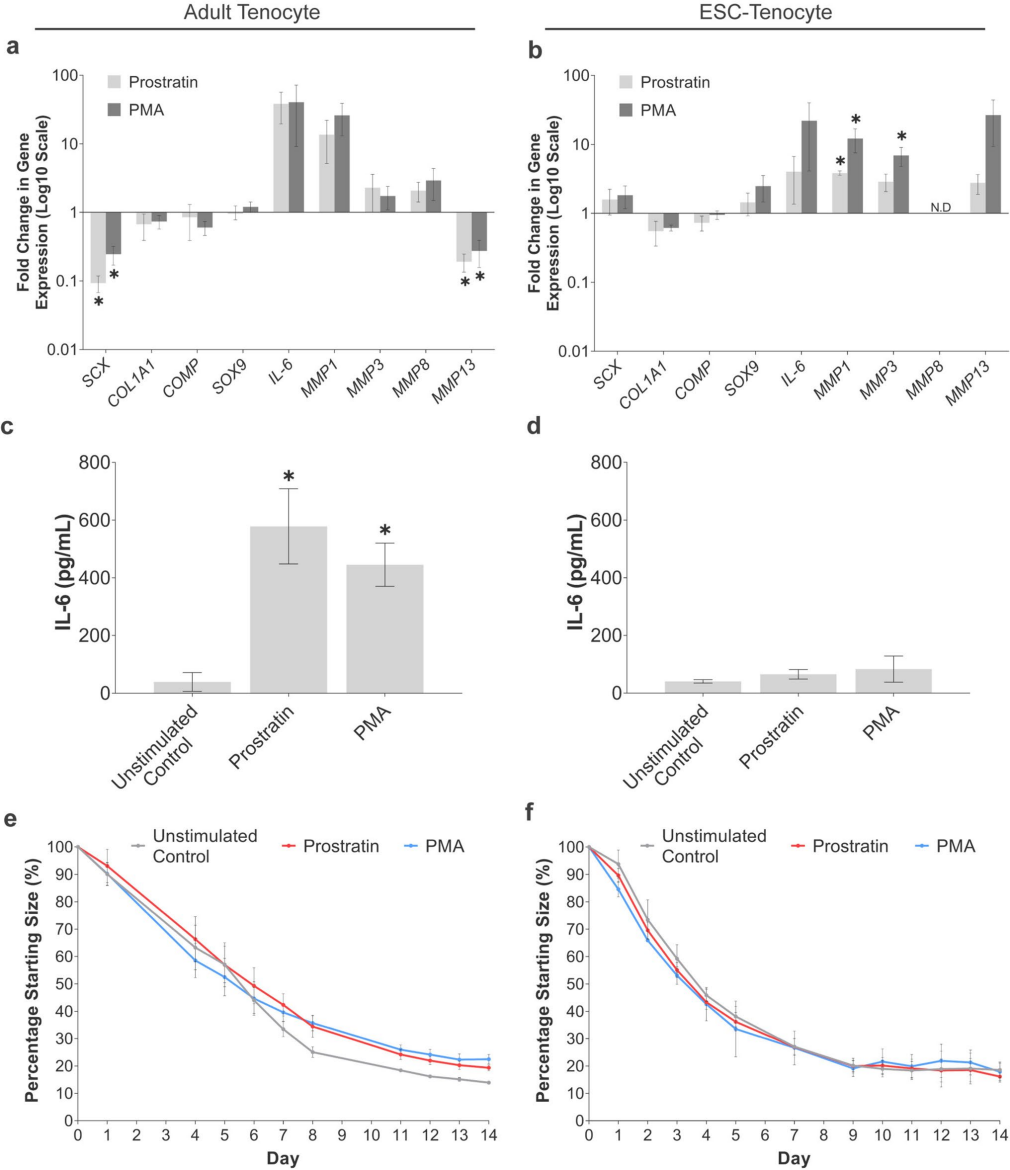
NF-κB activators cause changes to 2-D gene expression but not 3-D collagen gel contraction in adult and ESC-tenocytes. Fold change in gene expression of adult (**a**) and ESC-tenocytes (**b**) following 72 h stimulation with Prostratin and PMA compared to the unstimulated control. Secretion of IL-6 is significantly increased by Prostratin and PMA in adult tenocytes (**c**) but not in ESC-tenocytes (**d**). Error bars represent the S.E.M of three biological replicates. In 3-D culture, neither Prostratin or PMA inhibited the degree of collagen gel contraction by adult (**e**) or ESC-tenocytes (**f**). Error bars here represent the S.E.M of five biological replicates of adult tenocytes and three biological replicates of ESC-tenocytes. The asterisk (*) denotes a significant difference to the unstimulated control at *p* < 0.05. Adult tenocytes in these experiments were used between P2 and P8 and ESC-tenocytes between P9 and P31

Since we were able to induce NF-κB P65 nuclear translocation in ESC-tenocytes using Prostratin and PMA, we next investigated whether these NF-κB activators resulted in downstream changes to gene expression and 3-D collagen gel contraction. After 72 h stimulation, PMA and Prostratin had no significant effect on adult tenocyte viability (Supplementary Fig. 5) but significantly down-regulated expression of *SCX* compared to the unstimulated control (Fig. 7a). In contrast, the expression of *SCX* was unchanged in ESC-tenocytes (Fig. 7b). No significant effects on *COL1A1*, *COMP,* and *SOX9* were observed in either cell type. PMA and Prostratin affected *IL-6* and *MMP* gene expression in both cell types, generating substantial increases in *IL-6* and *MMP1* and a smaller, but significant, decrease in *MMP13* in the adult tenocytes. In the ESC-tenocytes, large increases in the expression of *IL-6*, *MMP3,* and *MMP13* were observed with PMA and slightly smaller increases with Prostratin. Not all the observed changes in gene expression were significant despite a consistent increase being observed in all replicates. The increase in *IL-6* gene expression in adult tenocytes was reflected at the protein level (Fig. 7c), however, no increase in IL-6 protein was observed in the ESC-tenocytes (Fig. 7d). Finally, Prostratin and PMA had no significant effect in inhibiting 3-D collagen gel contraction by adult or ESC-tenocytes (Fig. 7e and f).

### ESC-tenocyte Conditioned Media is Unable to Fully Rescue the Negative Effects of Inflammatory Cytokines on Adult Tenocytes

**Fig. 8:**
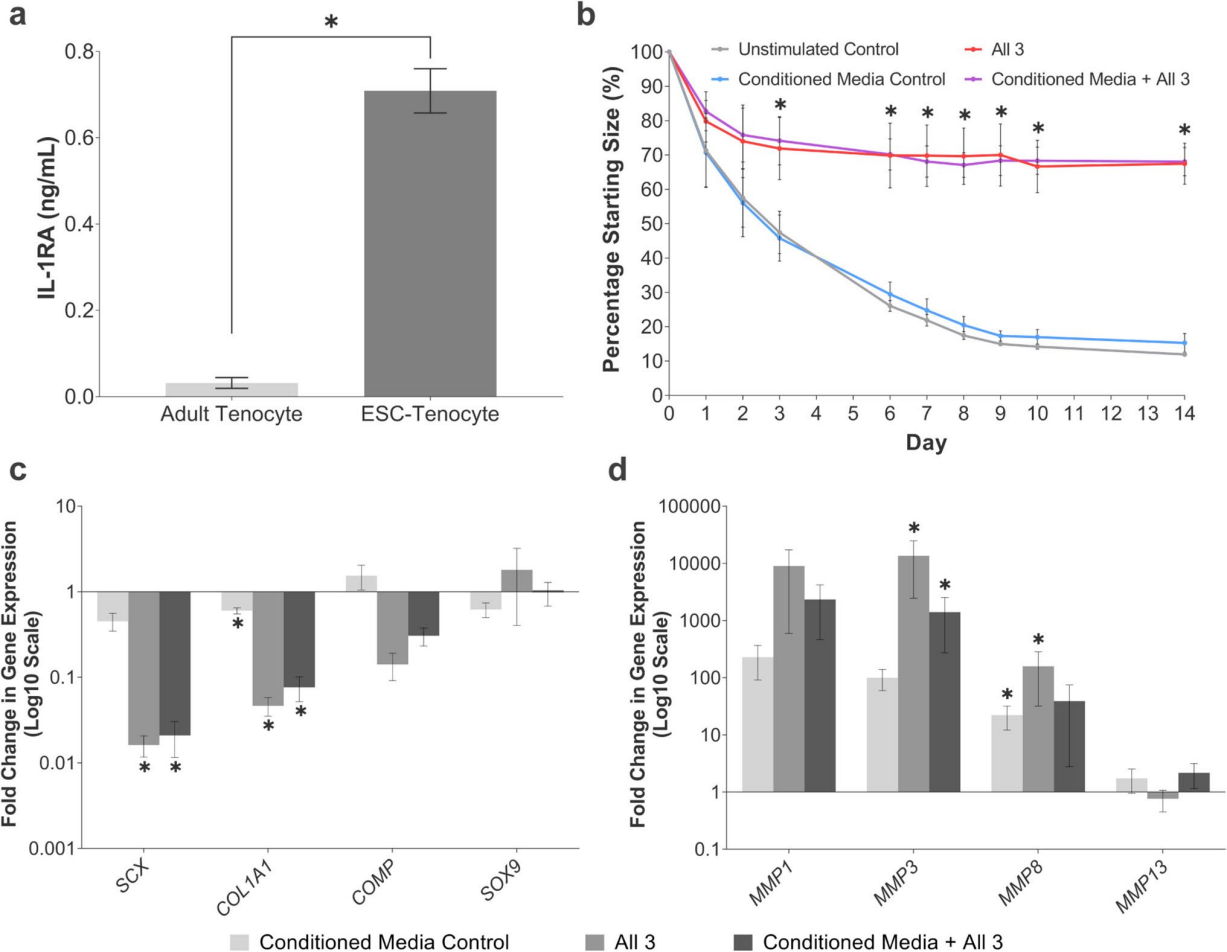
ESC-tenocytes are unable to release soluble factors which protect adult tenocytes from the combined effects of IFN-γ, TNFα, and IL-1β. (**a**) ESC-tenocytes express significantly more IL-1RA than adult tenocytes. The asterisk (*) indicates significant difference between indicated conditions at *p* < 0.05. Error bars represent the S.E.M of three biological replicates. (**b**) ESC-conditioned media was unable to rescue the adverse effects IFN-γ, TNFα, and IL-1β (All 3) have upon 3-D collagen gel contraction by adult tenocytes. The asterisk (*) indicates the degree of collagen gel contraction is significantly different to the unstimulated control (*p* < 0.05). Error bars are representative of the S.E.M of three biological replicates of adult tenocytes cultured with conditioned media from three biological replicates of ESC-tenocytes. Fold change in tendon associated (**c**) and *MMP* (**d**) gene expression in adult tenocytes stimulated with IFN-γ, TNFα, and IL-1β alone or in combination with ESC-conditioned media compared to the unstimulated control. ESC-conditioned media alone served as an additional control. The asterisk (*) indicates significant difference relative to the unstimulated control in growth media *p* < 0.05. Error bars are representative of the S.E.M of four biological replicates of adult tenocytes cultured with conditioned media from three biological replicates of ESC-tenocytes. Adult tenocytes in these experiments were used between P1 and P6 and ESC-tenocytes between P9 and P31

Previously we had shown that ESC-tenocytes express high levels of the gene encoding IL-1 receptor antagonist protein (IL-1RA) compared to adult tenocytes [34]. Here we confirm that this is reflected at the secreted protein level (Fig. 8a). However, in 3-D culture, conditioned media taken from ESC-tenocytes failed to rescue collagen gel contraction by adult tenocytes following stimulation with IL-1β, TNFα, and IFN-γ (Fig. 8b). Additionally, ESC-tenocyte conditioned media had little effect in rescuing tendon-associated gene expression in adult tenocytes following stimulation with all three cytokines in combination (Fig. 8c). There was a trend for ESC-conditioned media to rescue *MMP* expression, however, this was not significant for all genes investigated likely due to the variation in the fold increase between biological replicates (Fig. 8d). Interestingly, ESC-conditioned media alone caused adverse changes to gene expression, including upregulating the expression of *MMPs*.

## Discussion

Various mechanistic models have been proposed to advance our understanding of tendinopathy. These research models have determined that although moderate levels of pro-inflammatory cytokines are necessary to induce tissue repair, excessive inflammation is a key driver of scar tissue deposition leading to poor functional outcomes. Despite this, the molecular pathways underpinning tendinopathy are largely unknown, meaning successful targeted therapeutics have so far eluded the field.

Our previous work demonstrated that IL-1β has adverse consequences for equine adult tenocytes, whereas ESC-derived tenocytes appeared to be unaffected [34]. However, this study only examined a small number of candidate genes. Here, we demonstrate using RNA sequencing that unlike adult tenocytes [17], ESC-tenocytes express no DE genes following IL-1β stimulation after two weeks in 3-D culture. This attenuated immune response in ESC-tenocytes is likely attributed to a disparate expression of the IL-1β signalling receptors, with ESC-tenocytes expressing low levels of IL-1R1 and high levels of the decoy IL-1R2 and gene encoding IL-1RA (*IL-1RN*) [34]. However, it is unknown whether equine ESC-tenocytes will respond to other pro-inflammatory cytokines which are present during tendinopathy.

Extensive research has focused on the consequences of individual cytokines on tenocyte responses; but when used in combination, the effects of IFN-γ, TNFα, and IL-1β on adult tenocytes are synergistic and magnified [13]. Here, we demonstrate that equine ESC-tenocytes do not respond to a combination of these cytokines, evidenced by exhibiting minimal changes to tendon-associated and *MMP* gene expression, and demonstrating no inhibition of 3-D collagen gel contraction; a measure of cell-mediated matrix reorganisation [42, 43]. A similar finding has been shown in ESCs derived from mice (mESC) and their differentiated vascular cells, where these cells elicited no response to TNFα stimulation [44]. In contrast, mESC-derived fibroblasts have been shown to acquire the ability to respond to TNFα during the differentiation process [45]. Other investigators have reported similar results in several types of cells differentiated from human and mouse ESCs including cardiomyocytes and osteoblasts [46–48]. Nevertheless, it should be noted that despite these differentiated cells gaining the ability to respond to inflammatory cytokines, their level of responsiveness was significantly lower than that of their native differentiated counterparts [45]. Conflicting results between studies of different ESC-differentiated cell types suggests that the inflammatory response is a developmentally regulated process which can be influenced by both differentiation protocol and duration, and cell type.

The NF-κB family of transcription factors play an essential role in the regulation of a broad range of genes involved in various inflammation and immune responses [49]. Investigations have implicated the NF-κB pathway in the early phases of tendon regeneration and inhibiting this pathway improved flexor tendon healing in a murine model [50, 51]. Once activated, NF-κB dimers rapidly translocate into the nucleus where they bind to DNA and modulate transcription of target genes. Unlike adult tenocytes [13], ESC-tenocytes failed to activate the canonical NF-κB pathway following stimulation with IFN-γ, TNFα, and IL-1β. Furthermore, the expression of IL-6 was also unaffected by inflammatory stimulation in ESC-tenocytes. Since IL-6 secretion has been extensively documented as a downstream consequence of NF-κB activation [49, 52, 53], this supports our findings. Investigation into other inflammatory pathways demonstrated that these inflammatory cytokines had similar effects in ESC-tenocytes as they do in adult tenocytes, demonstrating little effect in the activation of JNK, but displaying significant upregulation of STAT1 signalling following stimulation with IFN-γ either alone or in combination with other cytokines. This finding was not surprising since ESC-tenocytes possess receptors for IFN-γ, and we have previously shown that IFN-γ has the ability to upregulate MHC I expression in ESC-tenocytes [36].

To elucidate the mechanisms which contribute to the differences in inflammatory response between ESC-tenocytes and adult tenocytes, we first examined the TNFα induced inflammatory cascade in further detail. The broad range of biological activities mediated by TNFα are facilitated through interaction with two distinct receptors: tumour necrosis factor receptor 1 (TNFR1; gene name: *TNFRSF1A*) and tumour necrosis factor receptor 2 (TNFR2; gene name: *TNFRSF1B*) [45]. A comparison of the transcriptomic profiles of adult and ESC-tenocytes revealed that adult tenocytes express significantly more *TNFRSF1A* than ESC-tenocytes. Some studies have suggested that absent or low-level expression of the TNFα receptors is a common feature of undifferentiated human and mouse ESCs, and likely contributes to their inability to activate a TNFα-induced inflammatory response [44, 45, 54, 55]. However, other studies have demonstrated similar expression levels pre- and post-differentiation of human ESCs, despite only observing NF-κB nuclear translocation following differentiation [48]. After further investigation, we were unable to find any significant differences in expression of TNFR1 and TNFR2 between adult and ESC-tenocytes at the protein level. In human umbilical vein endothelial cells, TNFR1 is primarily localised to the Golgi apparatus rather than the plasma membrane; a characteristic which makes them resistant to TNF-induced apoptosis [56, 57]. Since we determined the presence of the TNFα receptors using immunofluorescence on permeabilised cells and western blot analysis, we cannot make conclusions on the intracellular locations of the receptors in ESC-tenocytes. Therefore, future work should utilise confocal microscopy analysis of intact and permeabilised cells to determine if adult and ESC-tenocytes have disparate localisation of the TNFα receptors, which may account for their failure to respond to TNFα.

Comparison of the transcriptomic profiles of adult and ESC-tenocytes revealed that adult tenocytes consistently expressed higher levels of many of the NF-κB pathway signalling components, although this was not statistically significant for any gene examined. Interestingly, ESC-tenocytes expressed significantly higher levels of *NFKBIA* and *NFKBIE*. These genes encode for IκBα and IκBε respectively, which form part of a subunit IκB which acts to inhibit NF-κB directed transactivation of NF-κB via cytoplasmic retention of the REL proteins. Although this finding may explain why NF-κB is inactive in ESC-tenocytes, we also found that ESC-tenocytes express significantly more *IKBKB* which acts to phosphorylate the IκB complex leading to the activation of NF-κB. These contrasting results are representative of the literature, where the functionality of NF-κB signalling in other species of ESCs and their differentiated derivatives has been somewhat controversial. Some studies have postulated that the expression of NF-κB components is a critical determinant for activation of NF-κB signalling in ESCs and their derivatives [45, 58, 59]. Whereas others have found no association between the expression of NF-κB components and the ability of these cells to initiate activation of NF-κB signalling [47]. Future work using global and unbiased phospho-proteomics analysis is therefore warranted to determine how the expression of these NF-κB components at the protein level influences NF-κB activation.

To determine whether the NF-κB pathway was functional in ESC-tenocytes, we investigated other NF-κB stimuli and found that, as in adult tenocytes, NF-κB activation could be induced through stimulation with the pharmaceuticals PMA and Prostratin. Interestingly, although these NF-κB activators did result in changes to tendon-associated gene expression in the ESC-tenocytes, these changes did not follow the same expression pattern as that seen in adult tenocytes. In adult tenocytes, *SCX* was significantly down-regulated whereas no change was seen in ESC-tenocytes. *IL-6* and *MMP* expression were upregulated in both cell types, although only the upregulation of *IL-6* in adult tenocytes was reflected at the protein level. This may be due to the large biological variation in the results, meaning that while there were some strong trends, they were not significant for all genes examined. Alternatively, these results could suggest that ESC-tenocytes are less responsive to these NF-κB pharmaceuticals, but a limitation of our work is that we did not determine whether the NF-κB nuclear translocation we observed in the ESC-tenocytes correlates to an equivalent level of DNA binding as produced in the adult cells [13]. Chromatin immunoprecipitation (ChIP) experiments may be useful to determine if NF-κB has different target genes in the different cell types [60]. Neither PMA nor Prostratin had any effect on 3-D contraction in either cell type, suggesting they may not be as potent as IL-1β/TNFα in activating NF-κB, or that they do not activate alternative NF-κB proteins which we have not measured such as the transcriptional repressor P50 [13]. Both pharmaceuticals stimulate NF-κB signalling through the activation of Protein Kinase C (PKC). PKC is a family of serine/threonine kinases which play a critical role in cell signalling and regulation [61]. PKC is not unique to the NF-κB pathway, and upon activation can phosphorylate a variety of downstream targets and transcription factors including MAPK/ERK signalling which has been shown to contribute to the development of tendinopathy [62]. Future work to determine if this signalling pathway is also being activated by PMA and/or Prostratin in adult or ESC-tenocytes is therefore required.

Studies suggest the therapeutic benefits of some stem cells are due to various paracrine factors such as serum proteins, growth factors, hormones, ECM proteins, and proteases secreted by these cells [63]. Despite this, our recent work has demonstrated that cell secretome or “conditioned media” from equine BM-MSCs is unable to rescue the negative effects a combination of inflammatory cytokines has on adult tenocytes *in vitro* [13]. Here, we examined ESC-conditioned media and found like BM-MSCs it had little effect in rescuing tendon-associated gene expression or 3-D collagen gel contraction in adult tenocytes following cytokine stimulation. Nevertheless, we were able to demonstrate that the ESC-conditioned media could slightly rescue *MMP* expression. Interestingly, these results mimic the effects we have observed when adult tenocytes are cultured with IFN-γ, TNFα, and IL-1β in combination with exogenous IL-1RA [13]. Analysis of the ESC-conditioned media found that ESC-tenocytes express significantly more IL-1RA than adult tenocytes which is in accordance with our previous work [34] and global gene expression analysis (Supplementary Fig. 3). TNFR1 can also be secreted as a soluble form to inhibit TNFα signalling [64–66], however, we were unable to find an assay which cross-reacted with the equine protein (data not shown). Unlike BM-MSC conditioned media, the ESC-tenocyte conditioned media alone had a negative effect on *MMP* expression. This is likely due to the ESC-tenocytes depleting the nutrients from the growth media before being cultured with the adult tenocytes. To overcome this, future research could supplement the ESC-conditioned media with fresh serum and/or glucose prior to co-culture to determine whether this produces an enhanced rescue effect.

The biological implications of an attenuated immune response in ESCs and their differentiated progeny can be viewed from different perspectives of stem cell, developmental and immunological biology. It is logical that ESCs do not respond to inflammatory cytokines when any disturbance of the immunological balance in the uterus may lead to an increased risk of pregnancy complications. In fact, TNFα and IFN-γ are embryotoxic cytokines which hinder embryonic development [67, 68]. Therefore, it is likely that the diminished immune response seen here in ESC-tenocytes is inherited from undifferentiated ESCs, although we did not directly measure the effect of the inflammatory cytokines on the undifferentiated cells. This characteristic may also provide an advantage to ESC-derived cell-based therapies where persistent tissue inflammation is present, but the long-term fate and functionality of transplanted cells may be compromised if they cannot respond to subsequent infection. However, this was an *in vitro* study and the ESC-tenocytes may be unaffected by inflammation because the differentiation protocols we use do not promote the development of the innate immune system in the time frame studied [69]. Future work should consider monitoring the response of ESC-tenocytes to inflammatory cytokines over longer periods of differentiation *in vitro* and *in vivo*.

In conclusion, our research has shown that ESC-tenocytes fail to respond to a combination of IL-1β, TNFα, and IFN-γ. While we do not yet fully understand the molecular basis for the underdeveloped cytokine response in ESC-tenocytes, our data suggests that there may be deficiencies at multiple levels of the signalling pathways. Further clarification of these mechanisms will enable the future development of novel pharmaceuticals to protect endogenous, adult tenocytes from the negative effects of excessive inflammation to improve tissue repair and regeneration following injury.

### Supplementary Information

Below is the link to the electronic supplementary material.

Supplementary file1 (DOCX 971 KB)

## Data Availability

All relevant data are within the manuscript and its supplementary information files. The RNA sequencing data generated in this project is available through NCBI GEO (https://www.ncbi.nlm.nih.gov/geo/) under accession number GSE237821. Other RNA sequencing data referred to in the manuscript is available under accession numbers GSE221370 and GSE145029.
