# Supplementary material for: Equine Embryonic Stem Cell-Derived Tenocytes are Insensitive to a Combination of Inflammatory Cytokines and Have Distinct Molecular Responses Compared to Primary Tenocytes
Source: Stem Cell Rev Rep. 2024 Feb 24;20(4):1040–59. doi: 10.1007/s12015-024-10693-8 (PMC11087315; doi:10.1007/s12015-024-10693-8)
Supplement: Supplementary file 1 — Supplementary file1 (DOCX 971 KB) [file 12015_2024_10693_MOESM1_ESM.docx]

## **Stem Cell Reviews and Reports**

**Equine Embryonic Stem Cell-Derived Tenocytes are Insensitive to a Combination of Inflammatory Cytokines and Have Distinct Molecular Responses Compared to Primary Tenocytes**

**Emily J. Smith^1^**, Ross E. Beaumont^1^, Jayesh Dudhia^1^ and Deborah J. Guest^1^

^1^ Department of Clinical Sciences and Services, The Royal Veterinary College, Hawkshead Lane, North Mymms, Hatfield, Herts AL9 7TA, UK

Correspondence and requests for materials should be addressed to Emily J. Smith (email: [ejsmith@rvc.ac.uk](mailto:ejsmith@rvc.ac.uk)) or Deborah J. Guest (email: [djguest@rvc.ac.uk](mailto:djguest@rvc.ac.uk)).

# Supplementary Figures


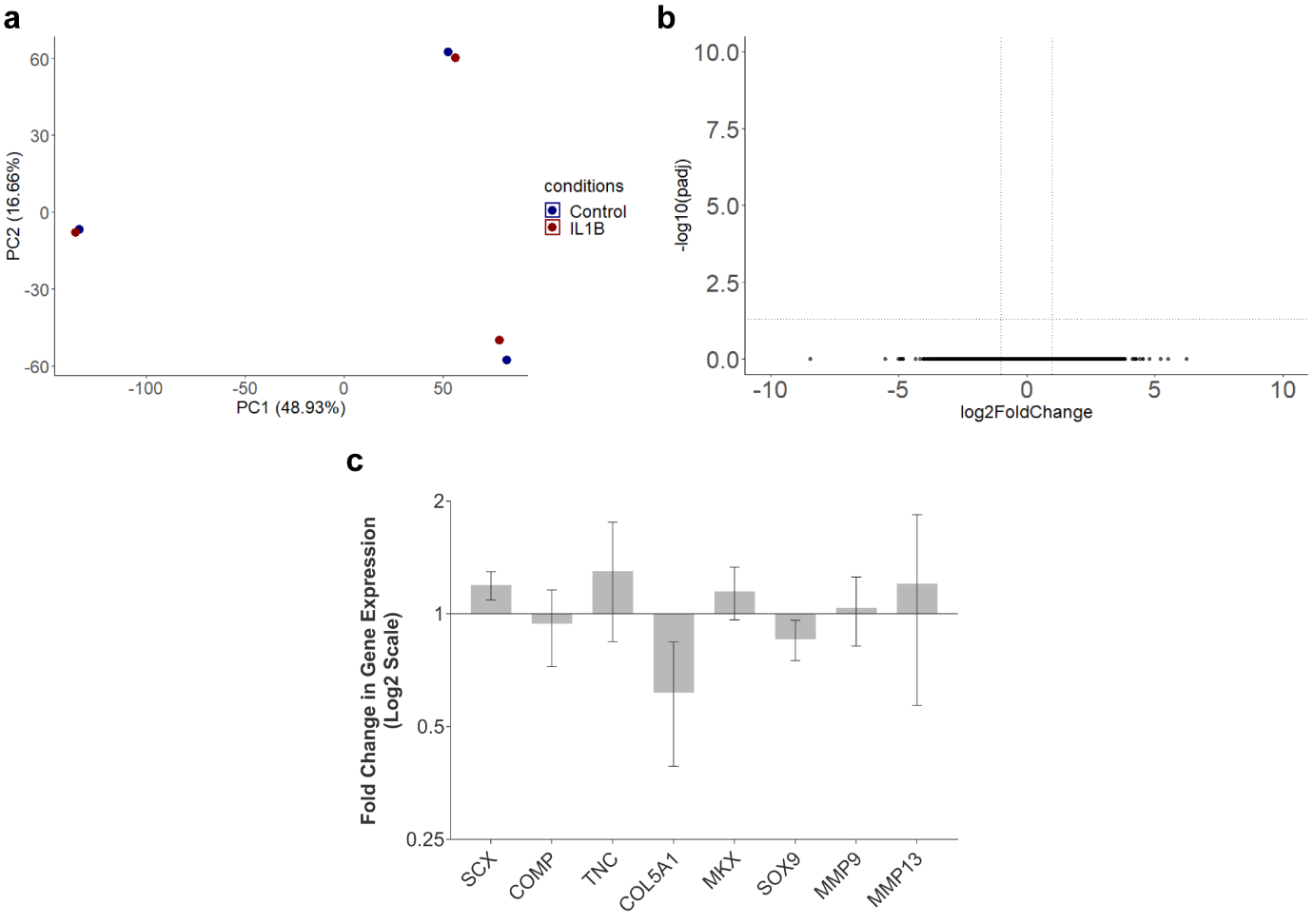
**Supplementary Fig. 1** (a) PCA of global gene expression profiles from three biological replicates of ESC-tenocytes in control (blue) and IL-1β (red) conditions. (b) Volcano plot demonstrating no genes were differentially expressed in ESC-tenocytes following stimulation with IL-1β. The x-axis represents the log2-fold change, and the y-axis depicts the -log 10 (adjusted *p* value). (c) qPCR validation of eight genes that undergo no significant change following ESC-tenocyte exposure to IL-1β but have been shown to be impacted by IL-1β in adult tenocytes [14]


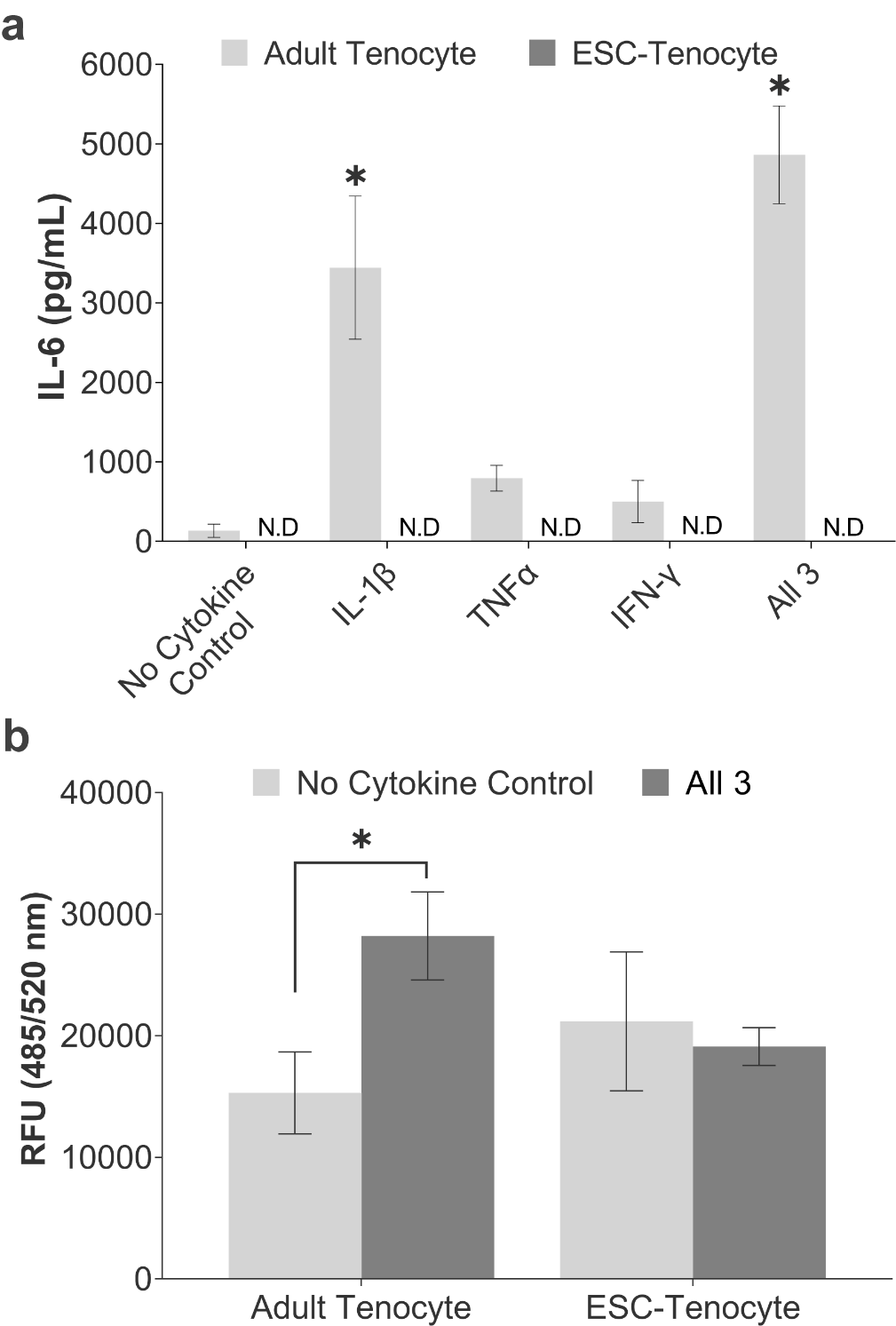


**Supplementary Fig. 2** Effect of inflammatory cytokine stimulation on protein secretion in ESC-tenocytes. (a) Secretion of IL-6 by ESC-tenocytes is unaffected by IFN-γ, TNFα, and/or IL-1β stimulation. Previously published adult data is shown as a comparison [13]. (b) Total MMP activity of adult and ESC-tenocytes treated with IFN-γ, TNFα, and IL-1β (all 3) for 1 h. Unstimulated cells served as a control. The asterisk (*) denotes a significant difference between indicated conditions at *p <* 0.05. Error bars represent the S.E.M of four biological replicates of adult tenocytes (P4 – P6) and three biological replicates of ESC-tenocytes (P14 – P20)


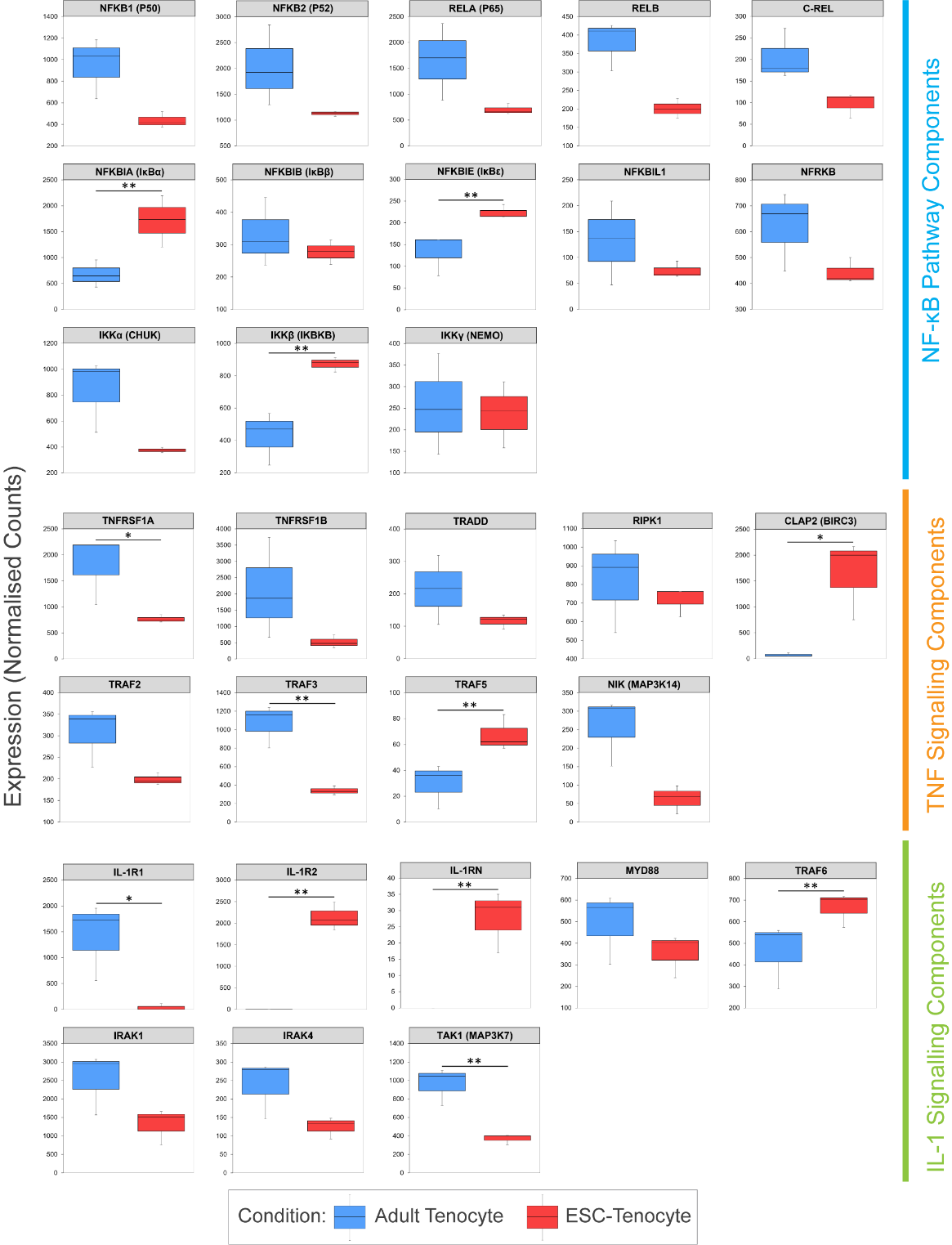
**Supplementary Fig. 3** Transcriptomic comparison of adult and ESC-tenocytes taken from publicly accessible data (Paterson *et al.*, 2020, GSE145029). Boxplots show gene expression of NF-κB pathway, TNF signalling, and IL-1 signalling components in unstimulated adult (blue) and ESC-tenocytes (red). Y-axis represents the gene expression (normalised counts). Significant differences are depicted by a single asterisk (*) for *p <* 0.05 and a double asterisk (**) for *p <* 0.01. Data is representative of three biological replicates of adult and ESC-tenocytes cultured in 3-D for 14 days

**
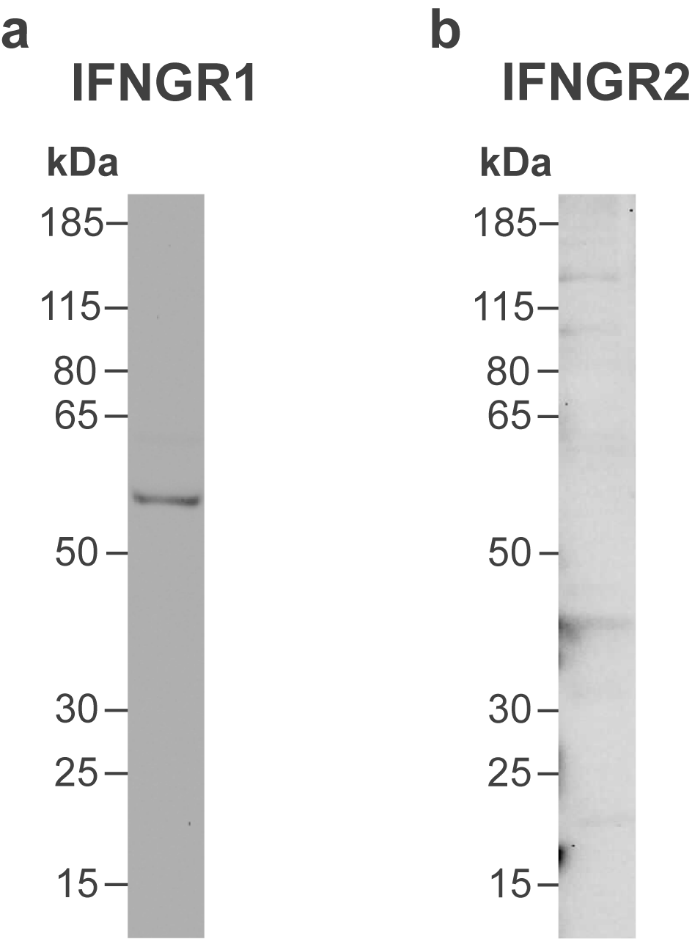
Supplementary Fig. 4** Western blot of primary antibodies. Western blotting showing binding of IFNGR1 (a) and IFNGR2 (b) antibodies to equine cytoplasmic protein from adult tenocytes and producing expected band sizes (IFNGR1 = 54 kDa, IFNGR2 = 38 kDa)


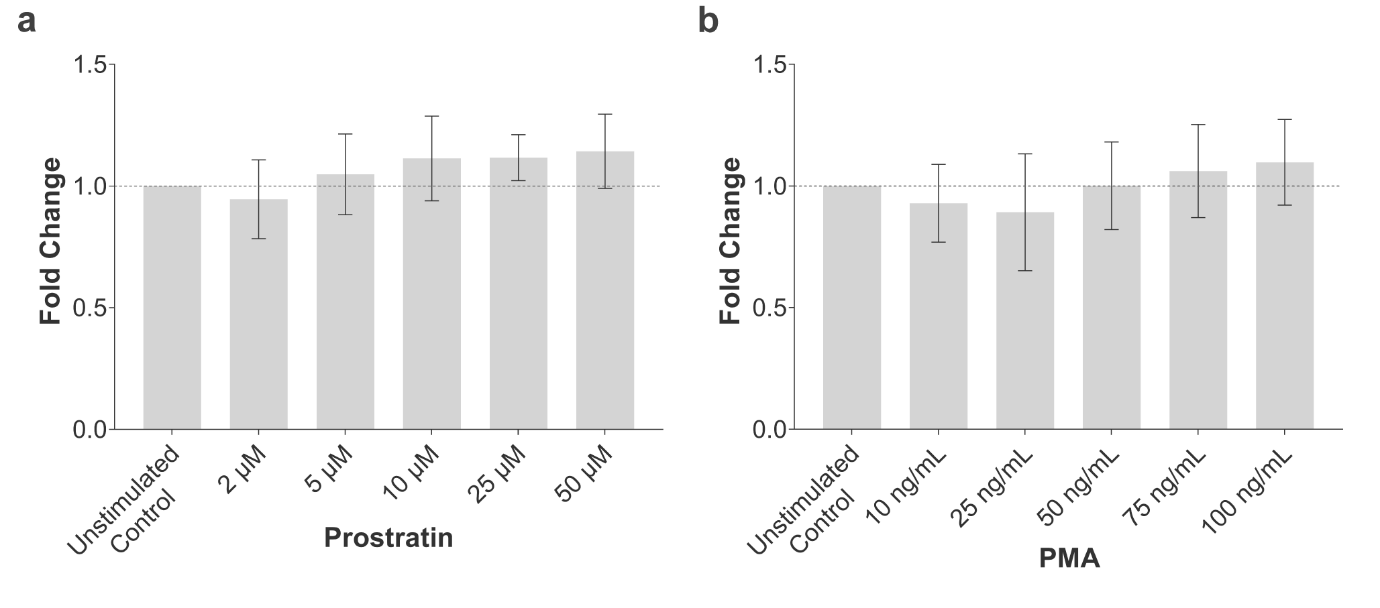
**Supplementary Fig. 5** Effects of Prostratin and PMA on cell viability in adult tenocytes. Prestoblue™ assay showing no significant effect of various concentrations of Prostratin (a) and PMA (b) on adult tenocyte cell viability following 72 hr stimulation. Data is presented as fold change in fluorescence compared to the unstimulated control. Error bars represent the S.E.M of three biological replicates of adult tenocytes. Cells in these experiments were between P4 and P8
